# Supplementary material for: Evolutionary Analysis and Functional Identification of Clock-Associated PSEUDO-RESPONSE REGULATOR (PRRs) Genes in the Flowering Regulation of Roses
Source: Int J Mol Sci. 2022 Jun 30;23(13):7335. doi: 10.3390/ijms23137335 (PMC9266954; doi:10.3390/ijms23137335)
Supplement: Supplementary file 1 [file ijms-23-07335-s001.zip › Supplementary Figures(1).pdf]

Supplementary Figures

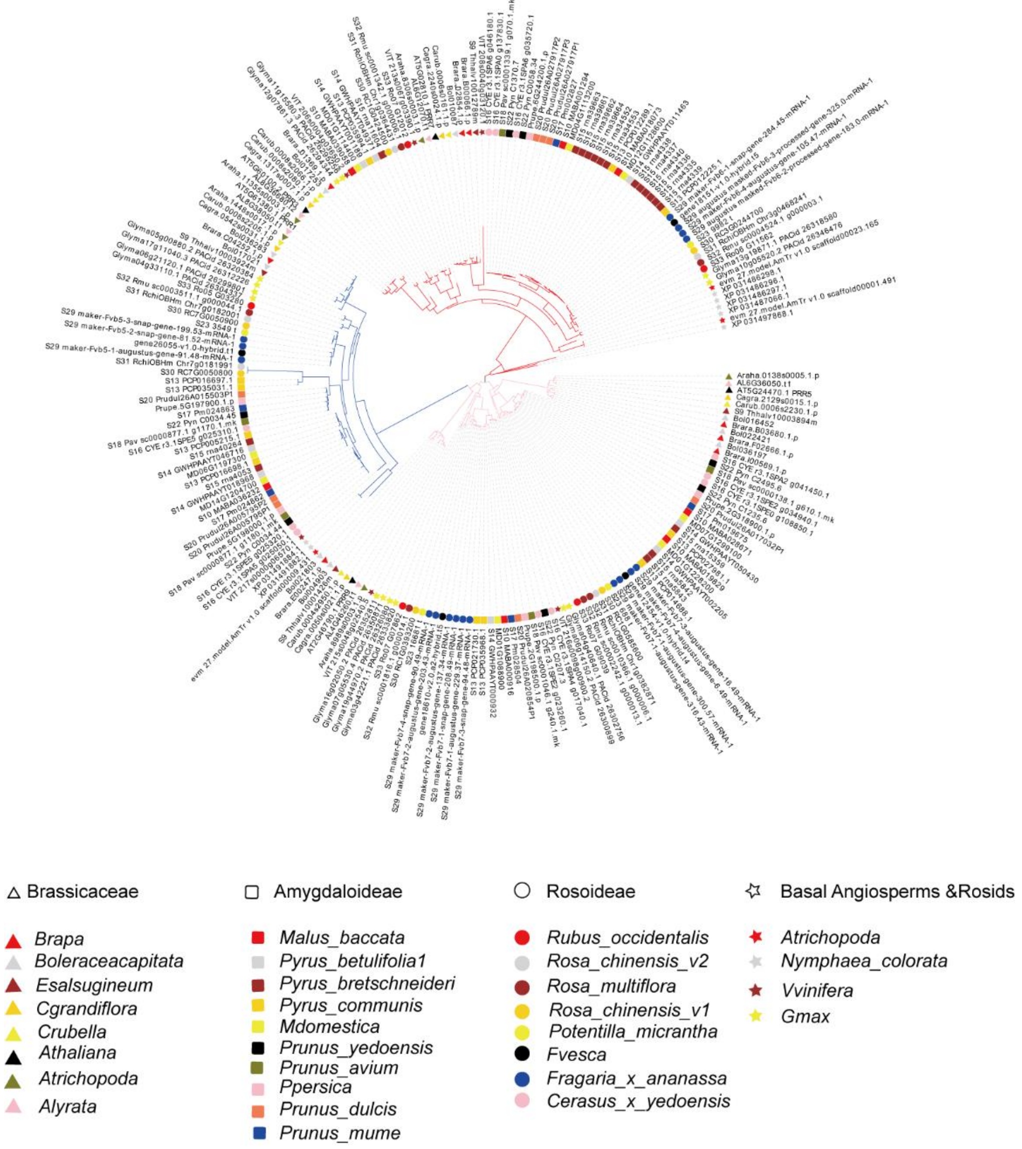

Figure S1. Phylogeny of clock genes from 2 basal angiosperm species, 18 Rosaceae species, 8 Brassicaceae species, and 2 Basal Rosids species. Different branch colors represent different clades.

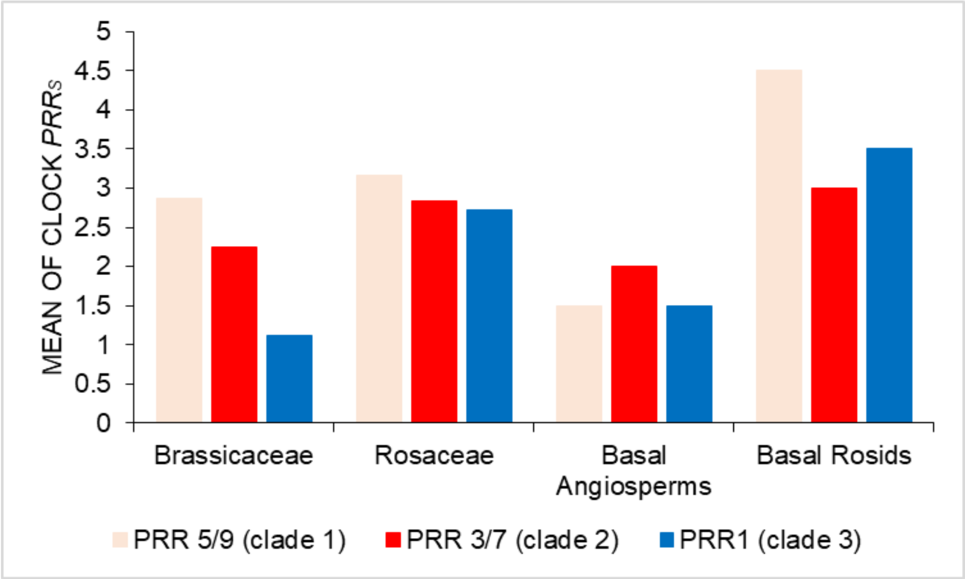

Figure S2. Mean values of the number of clock *PRRs* in each clade of 8 Brassicaceae species, 18 Rosaceae species, 2 basal angiosperm species, and 2 Basal Rosids species.

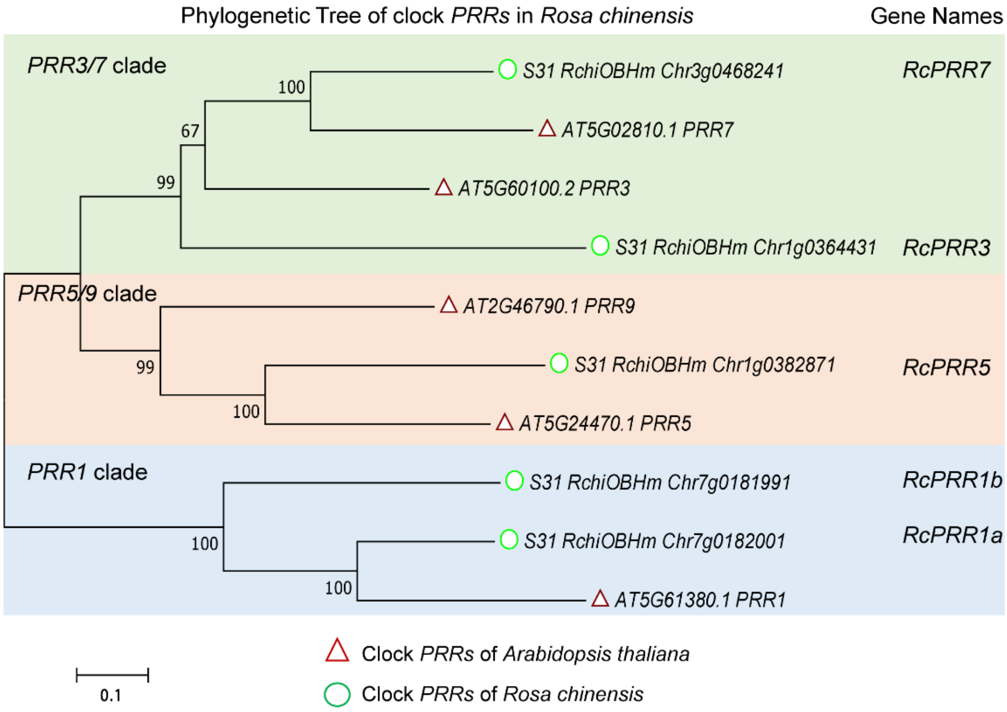

Figure S3. Phylogenetic tree of clock *PRRs* in *Rosa chinensis*.

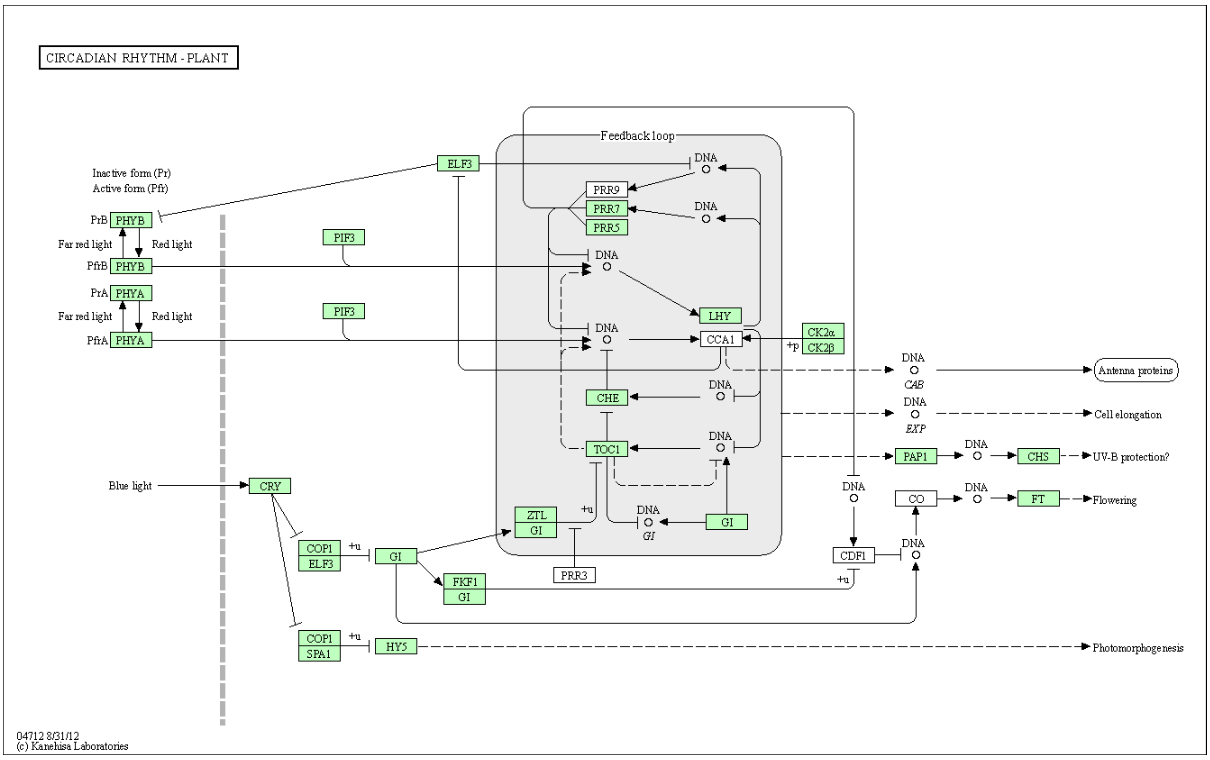

Figure S4. The pattern of clock *PRRs* genes for controlling flowering in the circadian rhythm KEGG pathway.
