# Supplementary material for: Evolutionary Analysis and Functional Identification of Clock-Associated PSEUDO-RESPONSE REGULATOR (PRRs) Genes in the Flowering Regulation of Roses
Source: Int J Mol Sci. 2022 Jun 30;23(13):7335. doi: 10.3390/ijms23137335 (PMC9266954; doi:10.3390/ijms23137335)
Supplement: Supplementary file 1 [file ijms-23-07335-s001.zip › Supplementary Tables(1).pdf]

Supplementary Tables

Table S1. Gene of the species that were not used in this study and deleted due to their partial/short sequences.

**Specie**	**Gene ID**
Arabidopsis lyrata	*AL8G36710.t2*
	AL6G52690.t1
Arabidopsis halleri	*Araha.20024s0001.1.p*
Fragaria ananassa	*S29_maker-Fvb5-1-snap-gene-91.65-mRNA-1*
	S29_maker-Fvb4-3-augustus-gene-246.20-mRNA-1
Fragaria vesca	*gene37163-v2.0.a2-hybrid.t2*
Cerasus yedoensis	*S16_CYE_r3.1SPE4_g004370.1*
Malus baccata	*S10_MABA036231*
Malus domestica	*MD14G1204500*
	MD07G1182300
Potentilla micrantha	*S23_8925_t*
Prunus persica	*Prupe.5G197800.1.p*
Prunus dulcis	*S20_Prudul26A024659P1*
Prunus mume	*S17_Pm024864*
Prunus yedoensis	*S22_Pyn_C2490.49*
	S22_Pyn_C0034.46
	S22_Pyn_C2268.2
Pyrus betulifolia	*S14_GWHPAAYT018966*
Pyrus bretschneideri	*S15_rna4054*
Rosa multiflora	*S32_Rmu_co8346237.1_g000001.1*
Vitis vinifera	*VIT_217s0000g06520.1*
	VIT_217s0000g06560.1

Table S2. Primers used in VIGS for amplification of genes fragments of *Rosa chinensis*.

**Gene Name**	**Primer**
RcPRR1a	F: TAGATAGGAGCAAGGTGAGG<br>R: AGACCTCATCCTGTGCCGAC
RcPRR5	F: AAGAACAATGGCGGAGGTGG<br>R: CCTTGCCTTCAGTATTTCCC

Table S3. Primers used for qPCR to check the relative expression of clock *PRRs* in *Rosa chinensis*.

Gene Name	Primers
RcPRR1a	F: ATTGCCTGCCCATGCTTCGT
	R: GCTGCGGCATGATTTGGCAT
RcPRR5	F: TGTTCAGGGCTGCTCCAGAGA
	R: ACGCTGCTCTGCAAGCTTCT
RcCO	F: GATACCACCGAGGACGGGTT
	R: CAGGACGCAGCCTCATCTTC
RcFT	F: TGCCGAGCTCTACAATCTTGG
	R: CATCACTTGAACCCGCCACT

Table S4. Basic Parameters of clock *PRRs* of *Rosa chinensis*.

Gene ID	Gene Name	Location	Chro	AA	CDS	Intron	MW/kDa	PI	Predicted subcellular localization
RchiOBHm_Chr7g0182001	*RcPRR1a*	3406338-3411730	7	535	1608	5	60139.19	5.77	Nucleus
RchiOBHm_Chr7g0181991	*RcPRR1b*	3400868-3402542	7	186	570	3	21258.23	4.48	Chloroplast
RchiOBHm_Chr1g0382871	*RcPRR5*	67470339-67474553	1	683	2086	7	75938.56	6.25	Nucleus
RchiOBHm_Chr1g0364431	*RcPRR3*	55607081-55612137	1	761	2324	7	83837.41	5.86	Nucleus
RchiOBHm_Chr3g0468241	*RcPRR7*	14299140-14304817	3	754	2302	8	82454.83	8.31	Mitochondrion
